# Age of First Exposure Does Not Relate to Post-Career Health in Former Professional American-Style Football Players

**DOI:** 10.1007/s40279-024-02062-9

**Published:** 2024-06-26

**Authors:** Douglas P. Terry, Rachel Grashow, Grant L. Iverson, Paula Atkeson, Ran Rotem, Shawn R. Eagle, Daniel H. Daneshvar, Scott L. Zuckerman, Ross D. Zafonte, Marc G. Weisskopf, Aaron Baggish

**Affiliations:** 1 Department of Neurological Surgery, Vanderbilt Sports Concussion Center, Vanderbilt University Medical Center, 1500 21st Ave South, Suite 4340, Village at Vanderbilt, Nashville, TN 37212 USA; 2 Harvard Medical School, Football Players Health Study at Harvard University, Boston, MA USA; 3 Department of Environmental Health, Harvard T. H. Chan School of Public Health, Boston, MA USA; 4 Department of Physical Medicine and Rehabilitation, Spaulding Rehabilitation Hospital, Charlestown, MA USA; 5 Department of Physical Medicine and Rehabilitation, Harvard Medical School, Charlestown, MA USA; 6 Department of Physical Medicine and Rehabilitation, Schoen Adams Research Institute at Spaulding Rehabilitation, Charlestown, MA USA; 7 Sports Concussion Program, Mass General for Children, Boston, MA USA; 8 Department of Neurological Surgery, University of Pittsburgh Medical Center, Pittsburgh, PA USA; 9 Cardiovascular Performance Program, Massachusetts General Hospital, Boston, MA USA; 10 Department of Cardiology, Lausanne University Hospital (CHUV) and Institute for Sport Science, University of Lausanne (ISSUL), Lausanne, Switzerland

## Abstract

**Objective:**

Prior studies examining small samples of symptomatic former professional football players suggest that earlier age of first exposure (AFE) to American football is associated with adverse later life health outcomes. This study examined a larger, more representative sample of former professional American football players to assess associations between AFE before age 12 (AFE < 12) and clinical outcomes compared with those who started at age 12 or older (AFE 12 +).

**Methods:**

Former professional American football players who completed a questionnaire were dichotomized into AFE < 12 and AFE 12 + . AFE groups were compared on outcomes including symptoms of depression and anxiety, perceived cognitive difficulties, neurobehavioral dysregulation, and self-reported health conditions (e.g., headaches, sleep apnea, hypertension, chronic pain, memory loss, dementia/Alzheimer’s disease, and others).

**Results:**

Among 4189 former professional football players (aged 52 ± 14 years, 39% self-reported as Black), univariable associations with negligible effect sizes were seen with AFE < 12, depressive symptoms (*p* = 0.03; *η*^2^ = 0.001), and anxiety-related symptoms (*p* = 0.02; *η*^2^ = 0.001) only. Multivariable models adjusting for age, race, body mass index, playing position, number of professional seasons, and past concussion burden revealed no significant relationships between AFE < 12 and any outcome. Linear and non-linear models examining AFE as a continuous variable showed similar null results.

**Conclusions:**

In a large cohort of former professional American-style football players, AFE was not independently associated with adverse later life outcomes. These findings are inconsistent with smaller studies of former professional football players. Studies examining AFE in professional football players may have limited utility and generalizability regarding policy implications for youth sports.

**Supplementary Information:**

The online version contains supplementary material available at 10.1007/s40279-024-02062-9.

## Key Points

**Table:** 

Prior studies suggested that starting to play football at younger ages (i.e., before 12 years old) may be associated with worse later-life outcomes (e.g., cognitive functioning, depression symptoms), but these studies had small sample sizes and all participants had self-reported cognitive, behavioral, and/or mood symptoms. Larger studies have not found this association.	
Questionnaire data provided by 4189 former American-style professional football players did not indicate that younger age of first exposure to football was significantly associated with any self-reported negative health outcomes when adjusting statistical models for other important variables.	
Future longitudinal cohort studies of youth football that further clarify the potentially harmful elements of youth football will assist parents and healthcare professionals in balancing the positive effects of football participation (e.g., cardiovascular conditioning, mentoring and community support, teamwork, self-esteem) and with the potential risks associated with play.	

## Introduction

In 2015, a study examining 42 former National Football League (NFL) players found that an age of first exposure (AFE) to football before the age of 12 was associated with lower neuropsychological test scores in participants who were approximately 52-years-old (standard deviation, 1.3) [1]. Accordingly, study authors suggested that exposure to repeated head impacts during a sensitive maturational period may be associated with later-life cognitive impairment. A number of authors have since identified methodological issues with this study related to sample size, limited generalizability to youth athletes, choice of the age 12 cutoff, retrospective nature of reporting AFE, and interpretation of neuropsychological test findings [2–5]. Subsequent findings by the same investigators suggest that AFE < 12 years is associated with greater apathy [6], worse depressive symptoms [6], and structural differences on brain scans (i.e., lower fractional anisotropy in the corpus collosum on diffusion tensor imaging [7] and smaller thalamic volumes on T1-weighted MRI scans [8]) compared with those with an AFE ≥ 12 in small samples of professional football players. Additionally, deceased football players from various levels of play who had neuropathological changes consistent with chronic traumatic encephalopathy neuropathologic change (CTE-NC) and an AFE < 12 had an earlier age of onset of cognitive and neurobehavioral symptoms than those with AFE ≥ 12; however, they did not have greater severity of CTE-NC [9]. These studies appear to have contributed to advocacy efforts for legislation banning and/or more tightly regulating youth tackle football [10, 11].

In contrast, several research studies have found no associations between AFE and long-term brain health outcomes among former living high school athletes [12–14], collegiate athletes [14–17], and professional football players [18, 19]. For instance, a small study (*n* = 45) of former professional football players did not show associations between pre-high school exposure to football and later-life neuroradiological, neurological, and neuropsychological outcome measures [19]. Additionally, two recent cohort studies respectively examined 3506 [18] and 1784 former professional football players [20]. One study found no associations between AFE < 12 and later-life prevalence of depression, anxiety, dementia, or mild cognitive impairment, though they found small effect size differences between earlier AFE and greater symptoms of cognitive dysfunction, anxiety, depression, and behavioral dyscontrol [20]. The other study did not show any associations between AFE and self-reported symptoms of cognitive dysfunction, depression, or anxiety [18]. Possible associations between early life football participation and later-life health problems remain incompletely understood.

We therefore undertook the present study to further examine relationships between AFE and long-term health in former professional football players. This effort builds on prior work by our group [18] by leveraging a cohort of former professional football players that has expanded from 3506 to 4189, incorporating additional health outcomes (e.g., emotional and behavioral dyscontrol, sleep apnea, high blood pressure, low testosterone), alternative football exposure variables (e.g., total years of football), and other determinants of health [e.g., body mass index (BMI)].

## Methods

### Participants

The Football Players Health Study (FPHS) is a transdisciplinary strategic initiative devoted to the health and wellbeing of former professional American-style football players who formerly played professional football after 1960 [21]. Using a Community-Based Participatory Research model, the FPHS regularly engages former professional football players and family members to guide research objectives, player recruitment, results translation, and dissemination of findings to former players, families, clinicians, researchers, and the general public. Starting in 2015, all eligible former players (those who signed a contract with a professional football league after 1960) received electronic and residential mail study invitations; contact information was supplied by the NFL Players Association. A follow-up survey invitation coupled to a financial incentive was similarly disseminated starting in 2019. This study has been approved by the Institutional Review Board at the Harvard T.H. Chan School of Public Health, protocol number IRB18-1365. The study was performed in accordance with the ethical standards as laid down in the 1964 Declaration of Helsinki and its later amendments.

### Measures

All data were collected as part of the initial survey unless specifically noted that they were extracted from the follow-up survey. Demographic factors (e.g., age, race, height, weight) were self-reported. AFE to football was assessed with the question “How old were you when you began to play organized football?” for which participants provided their age in years. Participants also reported the number of years they actively practiced/played professional football and their primary field position.

Psychological and cognitive health at the time of survey completion were assessed using validated self-report questionnaires. The Patient Health Questionnaire (PHQ-4) is a brief self-report scale that consists of a two-item depression scale (i.e., the PHQ-2) and a two-item anxiety scale (GAD-2) that assesses symptoms over the past two weeks [22], with higher scores indicating more symptoms. Screening positively for depression and anxiety was operationalized as total PHQ-2 ≥ 3 and GAD-7 ≥ 3, respectively. Two Quality of Life in Neurological Disorders (Neuro-QoL) scales [23] were used to assess perceived cognitive difficulties (Neuro-QoL Applied Cognition—Cognitive Concerns; V2.0 Short Form) and irritability (Neuro-QoL Emotional and Behavioral Dyscontrol; Short Form). Each questionnaire contained eight items (e.g., “I had trouble concentrating,” “I had trouble controlling my temper”). Participants rated the frequency of the difficulties over the past 7 days (e.g., “Never” to “Always”). For each questionnaire, individual items are summed and the questionnaire raw score was converted to a T score to understand how the participants compared to a standard reference sample with a mean score of 50 and standard deviation (SD) of 10, consistent with standard practice [24]. Worse perceived cognitive functioning is associated with lower raw and T scores, while worse irritability is associated with higher raw and T scores.

Participants also indicated if they were ever recommended/prescribed medication by a medical provider for the following conditions (yes/no): headaches, pain, anxiety, depression, memory loss, attention-deficit/hyperactivity disorder (ADHD), hypertension, and low testosterone. In addition, participants were asked if a health care provider had previously rendered a diagnosis for any of the following health conditions (yes/no): dementia/Alzheimer’s disease (AD), vascular dementia (follow-up only), dementia other than AD/vascular dementia (follow-up only), and chronic traumatic encephalopathy [25].

### Statistical Analyses

Consistent with prior studies [7, 20], AFE was examined as a continuous variable, as well as dichotomized to lower than 12 years old (AFE < 12) or 12 years or greater (AFE 12 +). For continuous demographic and outcome variables, Kruskal–Wallis rank sum tests were used to determine univariable statistical significance with accompanying effect size *η*^2^. Chi-squared tests were used to examine potential differences in proportions of the AFE groups for categorical variables, with effect size Cramer’s *V*. The *η*^2^ effect sizes were interpreted as: < 0.01, negligible; 0.01–0.05, small; 0.06–0.013, medium; and ≥ 0.14, large [26]. Cramer’s *V* [degrees of freedom (df) = 1] effect sizes were interpreted as follows: 0.00–0.09, negligible; 0.10–0.29, small; 0.30–0.49, medium; and ≥ 0.50, large [26].

A series of multivariable linear regressions estimated associations between dichotomized AFE (0 = AFE 12 + ; 1 = AFE < 12) and each outcome. Similar to prior studies [25, 27], covariates included age, race, body mass index (BMI), playing position, total number of professional seasons, and concussion signs and symptoms score quartile (an approximation of prior concussion burden based on the number of times they recalled clinical concussion signs/symptoms following head impacts during football) [27]. Generalized linear regressions were used to determine whether there were linear associations between continuous AFE and each outcome. To investigate whether there were significant non-linear relationships between continuous AFE and each outcome that would not be detected by standard linear regression models, we implemented penalized spline regression in generalized additive models. The application of a penalty for the addition of each knot reduces the likelihood of overfitting and balances the model fit with the data. Model diagnostics were run to identify model misspecification. All model types (AFE as binary, AFE as a continuous linear term, and AFE as a spline) were additionally run using only age and race as covariates.

No adjustments for multiple comparisons were performed to optimize the identification of all possible significant results at the accepted risk of type 1 statistical errors. Statistics were calculated using R Language for Statistical Computing [28].

## Results

Of 16,138 players who were sent invitations to participate in the survey, 4189 (26.0%) enrolled and completed all relevant data fields, and were broadly representative of all former professional ASF players by age, weight, height, and playing position (Zafonte et al. [21]). At the time of enrollment, age ranged from 24–89 (mean ± SD, 52 ± 14 years) and year of professional football debut ranged from 1952 to 2016. Demographic and univariate comparisons are shown in Table 1 and Supplementary Table 1. Roughly half of the cohort self-identified as white (57%) and 39% identified as Black. For select health outcomes (denoted with an asterisk in Table 1), follow-up data from 1980 participants were used (comparisons between retained and loss to follow up are shown in Supplementary Table 2).

**Table 1:** Demographics and health variables of sample, stratified by age of first exposure

	Total (*N* = 4189)	AFE < 12 (*n* = 1824)	AFE 12 + (*n* = 2365)	*p*	Effect size
*Age*				< 0.001	*η*^2^ = − 0.043 Small–medium
Mean (SD)	51.8 (14.4)	48.2 (13.1)	54.5 (14.8)		
Median (IQR)	52 (39, 63)	49 (37, 57)	56 (41, 66)		
*Race*				0.011	*V* = 0.052 Negligible
White	2376 (56.7%)	989 (54.2%)	1387 (58.6%)		
Black	1634 (39.0%)	763 (41.8%)	871 (36.8%)		
American Indian/Alaska Native, Asian, Native Hawaiian/Pacific Islander, or other	126 (3.0%)	52 (2.9%)	74 (3.1%)		
Missing	53 (1.3%)	20 (1.1%)	33 (1.4%)		
*First professional football year*				< 0.001	*η*^2^ = 0.05 Small–medium
Mean (SD)	1986 (14.9)	1990 (13.6)	1983 (15.2)		
Median (IQR)	1985 (1974, 1999)	1989 (1980, 2001)	1982 (1971, 1997)		
Missing (*n*)	27	16	11		
*Number of professional football seasons*				< 0.001	*η*^2^ = 0.006 Negligible
Mean (SD)	6.67 (3.88)	6.35 (3.88)	6.91 (3.86)		
Median (IQR)	6 (4, 9)	6 (3, 9)	6 (4, 10)		
*Total number years playing football*				< 0.001	*η*^2^ = 0.25 Large
Mean (SD)	17.2 (4.7)	19.8 (4.0)	15.2 (4.2)		
Median (IQR)	17 (14, 20)	19 (17, 22)	15 (12, 18)		
*Lineman status*				< 0.001	*V* = 0.12 Large
No	2769 (66.1%)	1323 (72.5%)	1446 (61.1%)		
Yes	1420 (33.9%)	501 (27.5%)	919 (38.9%)		
*Age of first exposure*				< 0.001	*η*^2^ = 0.73 Large
Mean (SD)	11.7 (3.17)	8.7 (1.65)	13.9 (1.96)		
Median (IQR)	12 (9, 14)	9 (8, 10)	14 (12, 15)		
Missing (*n*)	52	52	0		
*Concussion signs and symptoms score*				< 0.001	*η*^2^ = 0.004 Negligible
Mean (SD)	30.8 (27.2)	32.5 (27.6)	29.4 (26.8)		
Median (IQR)	23.0 (11, 44)	25 (12, 46)	22 (9, 41)		
Current BMI				0.004	*η*^2^ = 0.002 Negligible
Mean (SD)	31.3 (5.0)	31.0 (4.9)	31.5 (5.1)		
Median (IQR)	30.4 (27.8, 33.8)	30.1 (27.7, 33.5)	30.6 (27.6, 34.8)		
Missing (*n*)	30	17	13		
*Current depression severity (Total PHQ-2)*				0.03	*η*^2^ = 0.001 Negligible
Mean (SD)	1.40 (1.7)	1.46 (1.7)	1.35 (1.7)		
Median (IQR)	1 (0, 2)	1 (0, 2)	1 (0, 2)		
*Positive screen for depression (PHQ-2 ≥ 3)*				0.17	
No	3303 (78.8%)	1420 (77.9%)	1883 (79.6%)		*η*^2^ = 0.021 Negligible
Yes	886 (21.2%)	404 (22.1%)	482 (20.4%)		
*Current anxiety severity (Total GAD-2)*				.02	*η*^2^ = 0.001 Negligible
Mean (SD)	1.57 (1.7)	1.64 (1.7)	1.52 (1.7)		
Median (IQR)	1 (0, 2)	1 (0, 2)	1 (0, 2)		
*Positive screen for anxiety (GAD-2 ≥ 3)*				0.16	
No	3211 (76.7%)	1379 (75.6%)	1832 (77.5%)		*η*^2^ = 0.022 Negligible
Yes	978 (23.3%)	445 (24.4%)	533 (22.5%)		
*Neuro-QoL Applied Cognition—Cognitive Concerns (T Score)*				0.07	*η*^2^ = 0.001 Negligible
Mean (SD)	40.6 (9.8)	40.3 (9.7)	40.8 (9.9)		
Median (IQR)	40.3 (34.8, 46.8)	40.3 (34.1, 45.6)	41.1 (34.8, 46.8)		
Emotional and Behavioral Dyscontrol (T Score)*				0.02	*η*^2^ < 0.001 Negligible
Mean (SD)	48.2 (11.1)	48.7 (11.6)	47.9 (10.8)		
Median (IQR)	48.1 (39.9, 55.8)	49.4 (39.9, 57.0)	48.1 (39.9, 55.8)		
*Diagnosis from a healthcare provider*					
Chronic traumatic encephalopathy	121 (2.9%)	50 (2.7%)	71 (3.0%)	0.62	*V* = 0.007
Dementia/Alzheimer’s disease	147 (3.5%)	56 (3.1%)	91 (3.8%)	0.18	*V* = 0.021
Vascular dementia*	37 (1.9%)	13 (1.5%)	24 (2.1%)	0.37	*V* = 0.017
Other dementia*	108 (5.5%)	44 (5.2%)	64 (5.6%)	0.72	*V* = 0.006
*Recommended or prescribed medicine*					
Depression	368 (8.8%)	155 (8.5%)	213 (9.0%)	0.57	*V* = 0.002
Anxiety	411 (9.8%)	172 (9.4%)	239 (10.1%)	0.47	*V* = 0.007
Memory loss	664 (16.3%)	272 (15.3%)	392 (17.0%)	0.14	*V* = 0.022
High blood pressure	1550 (37.3%)	571 (31.7%)	979 (41.7%)	< 0.001	*V* = 0.103, Small
Pain	1151 (27.5%)	496 (27.2%)	655 (27.7%)	0.72	*V* = 0.005
ADHD/ADD	412 (10.1%)	194 (10.9%)	218 (9.5%)	0.16	*V* = 0.022
Sleep apnea	940 (22.4%)	376 (20.6%)	564 (23.8%)	0.01	*V* = 0.038, Negligible
Low testosterone	730 (17.9%)	285 (16.0%)	445 (19.3%)	0.006	*V* = 0.043, Negligible
Headaches	1016 (24.9%)	464 (26.1%)	552 (24.0%)	0.12	*V* = 0.024

ADHD/ADD, attention-deficit/hyperactivity disorder and attention deficit disorder; AFE, age of first exposure; *η*^2^, effect size for Kruskal–Wallis test (interpreted as: < 0.01, negligible; 0.01–0.05, small; 0.06–0.013, medium; ≥ 0.14, large); effect size *V* or Cramer’s *V*, effect size for chi-squared test (interpreted as: 0.00–0.09, negligible; 0.10–0.29, small; 0.30–0.49, medium; ≥ 0.50, large)

*IQR*, interquartile range; *GAD-2*, Generalized Anxiety Disorder-2; *Neuro-QoL*, Quality of Life in Neurological Disorders; *NFL*, National Football League; *PHQ-2*, Patient Health Questionnaire-2; *SD*, standard deviation

^*^These variables were assessed on the follow-up questionnaire (total *n* = 1980)

Compared with the AFE 12 + group (*n* = 2367; AFE = 14 ± 2 years), the AFE < 12 group (*n* = 1825; AFE = 9 ± 2 years) was younger by an average of 6 years, more likely to be Black (41.9% versus 36.8%), and they reported later calendar years of professional debut. In addition, participants in the AFE 12 + group played fewer total years of football than those in the AFE < 12 group and had slightly but statistically significantly shorter professional career durations. Compared with the AFE 12 + group, men in the AFE < 12 group reported greater prior concussion signs/symptoms.

Key univariable comparisons are summarized in Table 1. Compared with the AFE 12 + group, men in the AFE < 12 group reported modestly greater current symptoms of depression (*p* = 0.03, *η*^2^ = 0.001, negligible effect size) and anxiety (*p* = 0.02, *η*^2^ = 0.001, negligible effect size). There were no statistically significant differences on measures assessing cognitive difficulties (*p* = 0.06), emotional and behavioral dyscontrol (*p* = 0.14), or reporting of a CTE or dementia diagnosis. Similarly, there were no group differences regarding a prior medical recommendation for prescription of medication for depression, anxiety, memory loss, headaches, or ADHD/ADD. A lower proportion of the AFE < 12 group reported being recommended/prescribed medication or therapeutic intervention for high blood pressure (31.6% versus 41.7%, *p* < 0.001), sleep apnea (20.6% versus 23.9%, *p* = 0.01), and low testosterone (16.0% versus 19.3%, *p* = 0.006) compared with the AFE 12 + group.

**Fig. 1:**
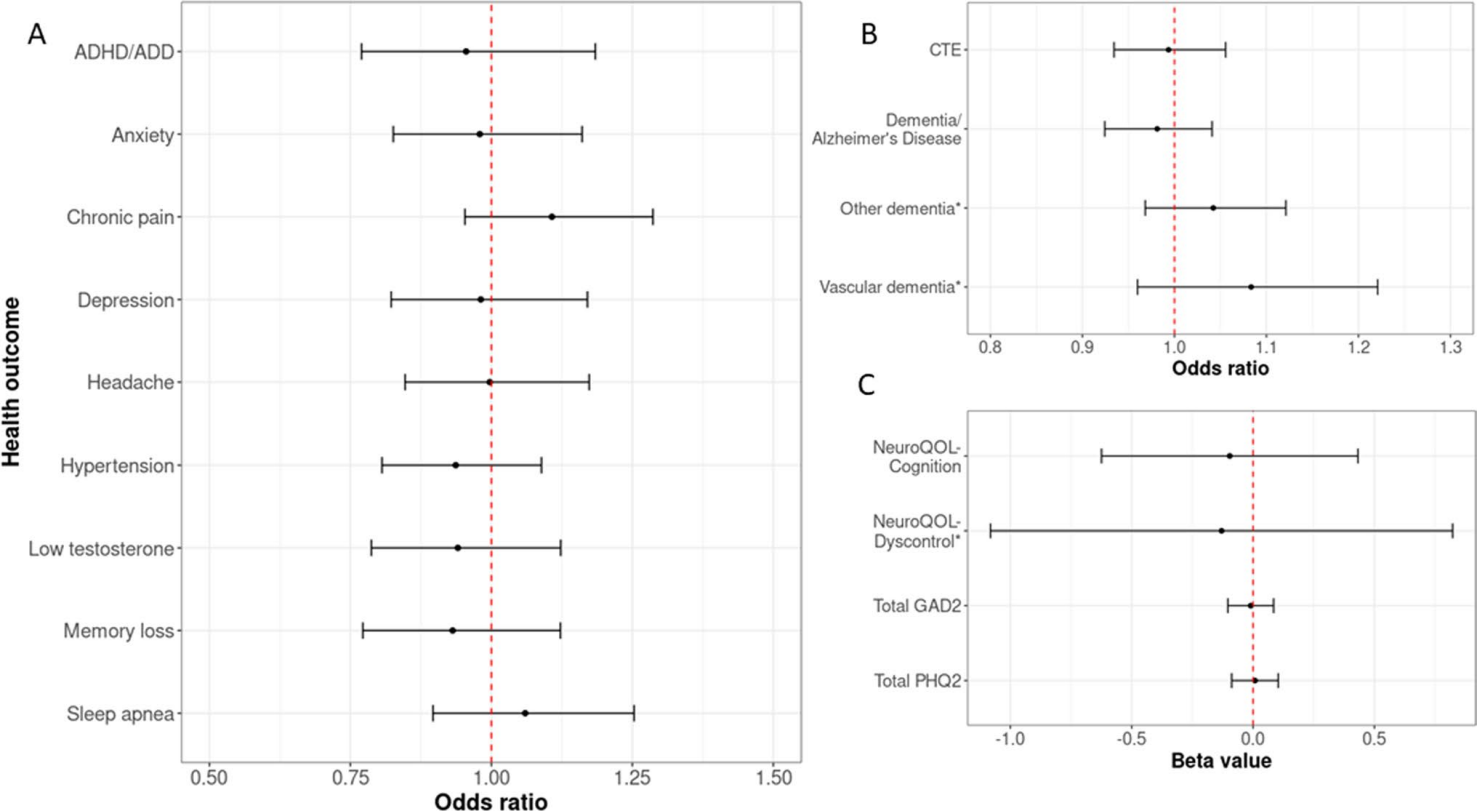
Associations between dichotomous age of first exposure per year and health outcome variables. Odds ratios and 95% confidence intervals from nondementia binary health outcomes are shown in A; odds ratios and 95% confidence intervals for dementia outcomes are shown in B; and C shows *β* estimates and 95% confidence intervals for continuous health outcomes. Note that each line represents a separate multiple regression/logistic regression model for age of first exposure (AFE) independent variable, such that AFE 12 + was the reference group (coded 0) and AFE < 12 was the experimental group (coded 1). Each regression model also included the following covariates: age at survey completion, race, current BMI, primary position (linemen versus not), number of professional seasons, and concussion signs and symptoms score quartile. *ADHD/ADD*, attention-deficit/hyperactivity disorder/attention deficit disorder; *CTE*, chronic traumatic encephalopathy; *GAD2-*, generalized anxiety disorder-2; *PHQ2*, patient health questionnaire-2; *Neuro-QoL*, Quality of Life in Neurological Disorders

A series of multivariable linear and logistic regressions examined whether AFE < 12 was associated with worse outcomes (Table 2, Fig. 1). Each model was designed to include covariates of age at survey completion, race, current BMI, primary field position (linemen versus not), number of professional seasons, and prior concussion signs and symptoms burden. The dichotomous AFE variable (AFE ≤ 12 versus AFE 12 +) was not significant in any of these 17 regression models (4 linear, 13 logistic). In similar analyses that included only age and race as covariates, results were unchanged (data not shown).

**Table 2:** Regressions results examining dichotomous age of first exposure on outcome variables

Dependent variable	*n*	Odds ratio/*β* value, [95% CI]	*p*
Neuro-QoL applied cognition—cognitive concerns	4095	*β* = − 0.10, [− 0.63, 0.43]	0.70
Neuro-QoL emotional and behavioral dyscontrol*	1928	*β* = − 0.16, [− 1.11, 0.79]	0.74
Current depression severity (total PHQ-2)	4096	β = 0.01, [− 0.09, 0.11]	0.82
Current anxiety severity (total GAD-2)	4096	β = − 0.01, [− 0.10, 0.09]	0.88
*Diagnosis from a healthcare provider*			
Dementia/Alzheimer’s disease	4101	OR = 1.39, [0.95, 2.05]	0.09
Chronic traumatic encephalopathy	4101	OR = 1.01, [0.68, 1.50]	0.97
Vascular dementia*	1944	OR = 1.13, [0.53, 2.41]	0.76
Other dementia*	1944	OR = 1.03, [0.65, 1.59]	0.94
Sleep apnea	4101	OR = 1.06, [0.90, 1.25]	0.47
*Recommended or prescribed medicine*			
Depression	4101	OR = 1.01, [0.80, 1.27]	0.97
Anxiety	4101	OR = 0.93, [0.74, 1.16]	0.50
Memory loss	4008	OR = 0.94, [0.78, 1.13]	0.51
High blood pressure	4072	OR = 0.94, [0.81, 1.08]	0.38
Chronic pain	4101	OR = 1.12, [0.97, 1.31]	0.13
ADHD/ADD	4013	OR = 0.97, [0.78, 1.20]	0.76
Low testosterone	4014	OR = 0.95, [0.79, 1.13]	0.54
Headache	4010	OR = 1.00, [0.85, 1.18]	1.00

An asterisk (*) indicates this was assessed on the follow-up questionnaire

*ADHD/ADD*, attention-deficit/hyperactivity disorder/attention deficit disorder; *CI*, confidence interval; *GAD-2*, generalized anxiety disorder-2; *Neuro-QoL*, Quality of Life in Neurological Disorders; *OR*, odds ratio; *PHQ-2*, Patient Health Questionnaire-2

Each line represents an independent multiple regression/logistic regression model for age of first exposure (AFE) independent variable, such that AFE 12 + was the reference group (coded 0) and AFE < 12 was the experimental group (coded 1). Each regression model also included the following covariates: age at survey completion, race, current BMI, primary position (linemen versus not), number of professional seasons, and concussion signs and symptoms score quartile

The same series of 17 linear and logistic regressions were repeated with AFE (in years) treated as a continuous variable in generalized linear regression models. Results remained the same in that AFE was not significantly associated with any adverse outcome (Supplementary Fig. 1). There were no significant non-linear relationships between continuous AFE and each outcome in spline regression models (Supplementary Table 3).

## Discussion

Prior studies examining possible associations between AFE to football and later-life psychological, cognitive, medical, and neurological symptoms in former professional athletes have yielded inconsistent findings. In this study, we examined a large cohort of former professional American-style football players to better understand associations between early AFE to football and a variety of outcomes including current psychological health symptoms, cognitive difficulties, neurobehavioral dysregulation, sleep apnea, low testosterone, hypertension, pain, diagnoses of dementia conditions, and health conditions such as anxiety, depression, headaches, and ADHD. Despite the finding that men in the AFE < 12 group had more cumulative football exposure over their athletic career than the AFE 12 + group, all adjusted outcome analyses showed no evidence that early AFE was independently associated with adverse later life health outcomes. Specifically, AFE examined both as a binary variable (i.e., cutoff at 12 years) and continuous variable (i.e., the exact AFE age reported) was not associated with any clinical outcomes in models that adjusted for demographic, football-related, and health-related factors. Further, in analyses that omitted potentially intermediate factors that could influence the association between AFE and long-term outcome (e.g., cumulative football exposure), outcomes were not related to AFE (data not shown).

Several prior studies that reported associations between AFE and adverse later-life outcomes were based on small sample sizes of overlapping participants (i.e., many participants’ data were used across several similar studies) and did not adjust for relevant covariates. In addition, some prior findings may have been influenced by sampling biases as study participants were required to have “self-reported complaints of cognitive, behavioral, and mood symptoms for at least the last 6 months” [1], which may limit the generalizability of these findings to asymptomatic former professional athletes. In this study, we attempted to overcome these limitations of prior work by examining a large sample size and by including numerous previously unexplored covariates in multivariable models such as current participant age, race, BMI, primary position (linemen versus not), number of professional seasons, and index of prior concussion signs/symptoms. Given that numerous risk factors and/or pathophysiological pathways may contribute to adverse clinical outcomes, we undertook comprehensive adjustment in our modeling to isolate the possible association with AFE. These methodological strengths may make the results of this study more rigorous, and more generalizable to all former professional football players compared with prior data derived from small samples of actively symptomatic men.

### Clinical and Policy Implications

The degree to which our findings can and should be considered in the formation of future athletic policy is beyond the scope of this paper. Given that the vast majority of youth athletes do not ultimately play professionally, studies that only examine former professional athletes lack the appropriate external validity to provide meaningful insights regarding the overall safety profile of youth tackle football. Nonetheless, our data show no link between AFE and post-career health among former professional football players, thereby suggesting that this exposure variable is less important than previously suggested and should not be used in isolation to determine youth sport policy as it relates to professional football players. Moreover, multiple studies involving other professional athletes, collegiate athletes, and former high school athletes have not found an association between earlier AFE and worse health outcomes [12, 13, 15–17].

### Limitations

There are several limitations of this study. First, data used to define both AFE and subsequent outcomes were self-reported by former athletes and may thus be subject to recall bias. Medical diagnoses were not validated on the basis of medical records, and perceived cognitive difficulties may not be related to objectively measured cognitive functioning [29]. Second, as participation in this survey was voluntary, the results may be influenced by ascertainment bias or healthy-worker bias. Third, the cross-sectional nature of our dataset renders our findings as associations and not causal relationships. Fourth, we did not control for multiple comparisons; doing so would have made some of the statistically significant univariate results nonsignificant. However, our inclusion of effect size measurements provides important clarification about the lack of clinical relevance of statistical significance given the large size of our sample. Fifth, this study specifically examined the association between starting tackle football before the age of 12 and risk of later-life cognitive, psychiatric, and other medical conditions among former NFL players. There may be other reasons to limit tackle football in youth, but these are beyond the scope of this study. Finally, our findings reflect the impact of AFE on former professional athletes and should be generalized with caution to people who played only youth, high school, or collegiate football, or other contact or collision sports. That said, null findings in this large sample may be relevant to athletes with far lesser football exposure and exposures to other contact and collision sports.

## Conclusions

Research on AFE to football has been used to advocate for policy regarding youth participation. However, these advocacy efforts have relied on findings from studies in former professional athletes that examined small sample sizes of symptomatic men and had other methodological limitations. Importantly, data from the present study found no meaningful associations between AFE and later life health status after comprehensive adjustment for a wide array of alternative football exposure variables. Accordingly, we saw no convincing signal that AFE of less than 12 years of age independently predicts later-life risk of adverse health outcomes among former NFL players. To date, cohort studies of former high school football players have not found an association between participation in football and later in life depression, suicidality, cognitive impairment, or neurological diseases [30]. Future longitudinal cohort studies of youth football might further clarify the potentially harmful elements of youth football so that children can experience the positive effects of football participation (e.g., cardiovascular conditioning, mentoring and community support, teamwork, self-esteem) and simultaneously reduce potential risks during this crucial developmental period of life.

## Supplementary Information

Below is the link to the electronic supplementary material.

Supplementary file1 (PDF 238 kb)

## Declarations

### Availability of Data and Material

Due to the high profile nature of the participants in this study, data are not available at this time.
