# Supplementary material for: Age of First Exposure Does Not Relate to Post-Career Health in Former Professional American-Style Football Players
Source: Sports Med. 2024 Jun 26;54(10):2675–84. doi: 10.1007/s40279-024-02062-9 (PMC11467122; doi:10.1007/s40279-024-02062-9)
Supplement: Supplementary file 1 — Supplementary file1 (PDF 238 kb) [file 40279_2024_2062_MOESM1_ESM.pdf]

Online Supplemental Material.

Age of First Exposure Does Not Relate to Post-Career Health in Former Professional American-Style Football Players

Authors

Douglas P. Terry, PhD*[1]
Rachel Grashow, PhD, MS*[2,3]
Grant L. Iverson, PhD[4,5,6,7]
Paula Atkeson, MPH[2]
Ran Rotem, PhD[3]
Shawn R. Eagle, PhD[8]
Daniel H. Daneshvar, MD, PhD[4]
Scott L. Zuckerman, MD, MPH[1]
Ross D. Zafonte, DO[2,4,5]
Marc G. Weisskopf, PhD[2,3]
Aaron Baggish, MD[2,9,10]

Affiliations

[1]Vanderbilt Sports Concussion Center, Department of Neurological Surgery, Vanderbilt University Medical Center, Nashville, TN, USA
[2]Football Players Health Study at Harvard University, Harvard Medical School, Boston, MA
[3]Department of Environmental Health, Harvard T. H. Chan School of Public Health, Boston, MA
[4]Department of Physical Medicine and Rehabilitation, Spaulding Rehabilitation Hospital, Charlestown, MA, USA
[5]Department of Physical Medicine and Rehabilitation, Harvard Medical School, Charlestown, MA, USA
[6]Department of Physical Medicine and Rehabilitation, Schoen Adams Research Institute at Spaulding Rehabilitation, Charlestown, MA, USA
[7]Sports Concussion Program, Mass General for Children, Boston, MA, USA
[8]Department of Neurological Surgery, University of Pittsburgh Medical Center, Pittsburgh, PA, USA
[9]Cardiovascular Performance Program, Massachusetts General Hospital, Boston, MA USA
[10]Department of Cardiology, Lausanne University Hospital (CHUV) and Institute for Sport Science, University of Lausanne (ISSUL), Lausanne, Switzerland

*denotes shared first authorship.

Supplemental Table 1. Participant playing positions.

	Total (N=4,192)	AFE<12 (n=1,825)	AFE 12+ (n=2,367)	p
**Main Position**				<.001
Defensive Back	606 (14.5%)	320 (17.5%)	286 (12.1%)	
Defensive Line	528 (12.6%)	184 (10.1%)	344 (14.5%)	
Kicker	130 (3.1%)	35 (1.9%)	95 (4.0%)	
Linebacker	641 (15.3%)	306 (16.8%)	335 (14.2%)	
Offensive Line	892 (21.3%)	317 (17.4%)	575 (24.3%)	
Quarterback	195 (4.7%)	92 (5.0%)	103 (4.4%)	
Running Back	396 (9.4%)	196 (10.7%)	200 (8.4%)	
Special Teams	34 (0.8%)	16 (0.9%)	18 (0.8%)	
Tight End	325 (7.8%)	139 (7.6%)	186 (7.9%)	
Wide Receiver	445 (10.6%)	220 (12.1%)	225 (9.5%)	

Note. AFE=age of first exposure.

Supplementary Table 2. Demographic and football exposure variables stratified by those who participated in the baseline survey only compared to those who provided follow up data.

	**Total (N=4,189)**	**Baseline only (N=2,209)**	**Follow-up (N=1,980)**	**p**
**Age at baseline**				<0.01
Mean (SD)	51.8 (14.4)	50.77 (14.96)	52.91 (13.72)	
Min ; Max	24.0 - 89.0	24.0 - 87.0	25.0 - 89.0	
Median (Q1, Q3)	52.0 (39.0, 63.0)	50.0 (38.0, 62.0)	54.0 (42.0, 64.0)	
**Race**				<0.01
White	2376 (56.7%)	1073 (48.6%)	1303 (65.8%)	
Black	1634 (39.0%)	1039 (47.0%)	595 (30.1%)	
Missing	53 (1.3%)	29 (1.3%)	24 (1.2%)	
American Indian/Alaska Native, Asian, Native Hawaiian/Pacific Islander, or Other	126 (3.0%)	68 (3.1%)	58 (2.9%)	
**First Professional Football Year**				<0.01
Mean (SD)	1986 (14.9)	1987 (15.5)	1984 (14.1)	
Min ; Max	1952 - 2016	1952 - 2016	1952 - 2016	
Median (Q1, Q3)	1985 (1974, 1999)	1988 (1975, 2001)	1983 (1973, 1996)	
N-Miss	27	22	5	
**Number of professional seasons**				0.217
Mean (SD)	6.7 (3.9)	6.7 (3.8)	6.6 (3.9)	
Min ; Max	0.0 - 26.0	0.0 - 25.0	1.0 - 26.0	
Median (Q1, Q3)	6.0 (4.0, 9.0)	6.0 (4.0, 9.0)	6.0 (4.0, 9.0)	
**Total Number Years Playing Football**				<0.01
Mean (SD)	17.2 (4.7)	17.5 (4.8)	16.9 (4.6)	
Min ; Max	1.0 - 45.0	1.0 - 45.0	1.0 - 39.0	
Median (Q1, Q3)	17.0 (14.0, 20.0)	17.0 (14.0, 21.0)	17.0 (14.0, 20.0)	
N-Miss	90	52	38	
**Age of First Exposure**				0.083
Mean (SD)	11.68 (3.17)	11.6 (3.3)	11.8 (3.1)	
Min ; Max	3.0 - 26.0	4.0 - 26.0	3.0 - 24.0	
Median (Q1, Q3)	12.0 (9.0, 14.0)	12.0 (9.0, 14.0)	12.0 (10.0, 14.0)	
N-Miss	52	24	28	
**Concussion Signs and Symptoms Score**				<0.01
Mean (SD)	30.7 (27.2)	32.6 (28.3)	28.7 (25.7)	
Min ; Max	0.0 - 130.0	0.0 - 130.0	0.0 - 130.0	
Median (Q1, Q3)	23.0 (11, 44)	25 (11.0, 47.0)	21.0 (10.50, 40.0)	
N-Miss	91	55	36	
**Lineman Status**				0.244
No	2769 (66.1%)	1478 (66.9%)	1291 (65.2%)	
Yes	1420 (33.9%)	731 (33.1%)	689 (34.8%)	

Supplementary Table 3. Spline term results for continuous per year AFE in generalized additive models of binary and continuous health outcomes.

	GAM Model		GAM diagnostics			
**Binary Outcomes**	**EDF**	**p-value**	**K'**	**EDF**	**k-index**	**p-value**
Health Conditions						
ADHD/ADD	1.00	.44	8	1.00	0.96	.88
Anxiety	5.17	.11	5	6.03	9.87	.13
Chronic pain	1.55	.45	8	1.55	0.95	.02
Depression	8.71	.21	23	8.71	0.94	.02
Headache	4.95	.20	8	4.95	0.98	.28
Hypertension	1.00	.37	8	1.00	0.99	.31
Low testosterone	1.85	.37	9	1.85	0.94	.06
Memory loss	2.69	.06	8	2.69	0.99	.96
Sleep apnea	2.81	.12	9	2.81	0.96	.10
Neurological Conditions						
Chronic Traumatic Encephalopathy	3.03	.35	9	3.03	0.92	.32
Dementia/Alzheimer's Disease	1.24	.66	8	1.24	0.94	.60
Other dementia	2.51	.20	9	2.51	0.93	.29
Vascular dementia	2.06	.11	9	2.06	0.91	.10
Continuous Outcomes						
NeuroQoL-Cognition	3.40	.30	9	3.4	0.99	.20
NeuroQoL-Dyscontrol	1.00	.33	9	1.00	0.99	.36
Total GAD2	2.90	.21	9	2.90	0.97	.07
Total PHQ2	2.51	.28	9	2.57	0.96	.02

Note. Each line represents a separate multiple regression/logistic regression model for age of first exposure (AFE) as a spline term. Each regression model also included the following covariates: age at survey completion; race; current body mass index (BMI); primary position (linemen vs. not); number of professional seasons; concussion symptom score quartile. ADHD/ADD=Attention-Deficit/Hyperactivity Disorder/Attention Deficit Disorder; GAD2=Generalized Anxiety Disorder-2; Neuro-QoL=Quality of Life in Neurological Disorders; PHQ2=Patient Health Questionnaire-2.

Supplementary Figure

Supplementary Figure 1. Associations Between Continuous Age of First Exposure and the Outcome Variables.

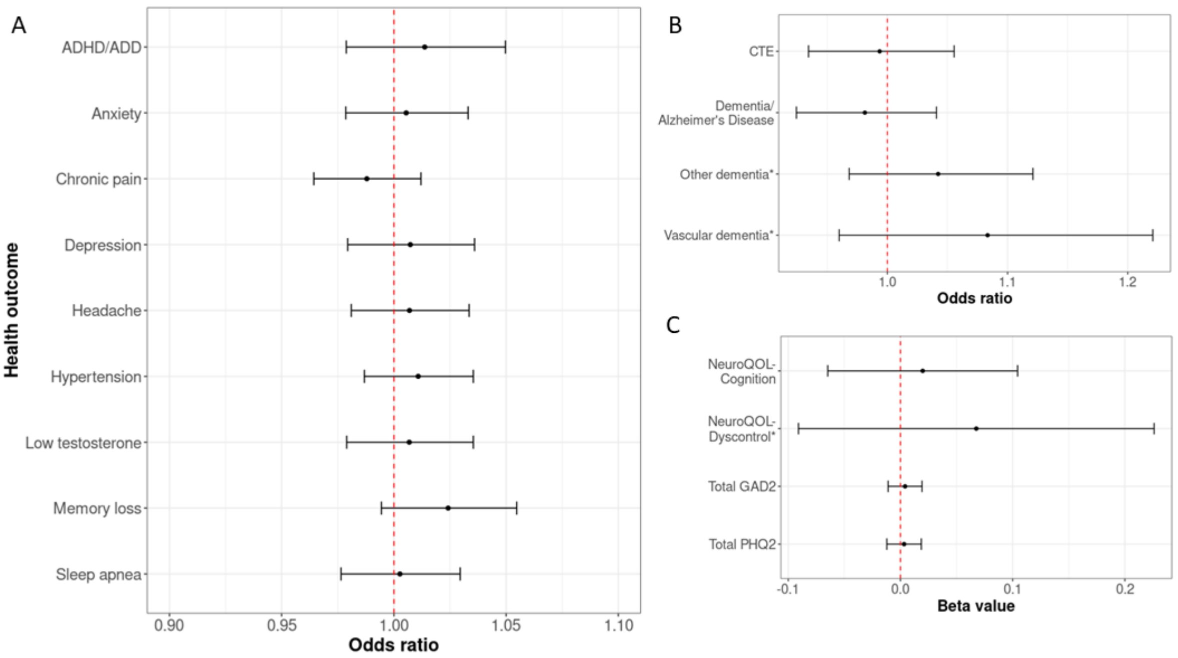

Odds ratios and 95% confidence intervals from non-dementia binary health outcomes are shown in A; odds ratios and 95% confidence intervals for dementia outcomes are shown in B; and C shows β estimates and 95% confidence intervals for continuous health outcomes. Note. Each line represents a separate multiple regression/logistic regression model for age of first exposure (AFE) as a continuous variable. Each regression model also included the following covariates: age at survey completion; race; current body mass index (BMI); primary position (linemen vs. not); number of professional seasons; concussion symptom score quartile. ADHD/ADD=Attention-Deficit/Hyperactivity Disorder/Attention Deficit Disorder; CTE=Chronic Traumatic Encephalopathy; GAD2=Generalized Anxiety Disorder-2; Neuro-QoL=Quality of Life in Neurological Disorders; PHQ2=Patient Health Questionnaire-2.
